# A signature-based method for indexing cell cycle phase distribution from microarray profiles

**DOI:** 10.1186/1471-2164-10-137

**Published:** 2009-03-30

**Authors:** Hideaki Mizuno, Yoshito Nakanishi, Nobuya Ishii, Akinori Sarai, Kunio Kitada

**Affiliations:** 1 Kamakura Research Laboratories, Chugai Pharmaceutical Co Ltd, Kamakura, Kanagawa, Japan; 2 Department of Biosciences and Bioinformatics, Kyushu Institute of Technology, Iizuka, Fukuoka, Japan

## Abstract

**Background:**

The cell cycle machinery interprets oncogenic signals and reflects the biology of cancers. To date, various methods for cell cycle phase estimation such as mitotic index, S phase fraction, and immunohistochemistry have provided valuable information on cancers (e.g. proliferation rate). However, those methods rely on one or few measurements and the scope of the information is limited. There is a need for more systematic cell cycle analysis methods.

**Results:**

We developed a signature-based method for indexing cell cycle phase distribution from microarray profiles under consideration of cycling and non-cycling cells. A cell cycle signature masterset, composed of genes which express preferentially in cycling cells and in a cell cycle-regulated manner, was created to index the proportion of cycling cells in the sample. Cell cycle signature subsets, composed of genes whose expressions peak at specific stages of the cell cycle, were also created to index the proportion of cells in the corresponding stages. The method was validated using cell cycle datasets and quiescence-induced cell datasets. Analyses of a mouse tumor model dataset and human breast cancer datasets revealed variations in the proportion of cycling cells. When the influence of non-cycling cells was taken into account, "buried" cell cycle phase distributions were depicted that were oncogenic-event specific in the mouse tumor model dataset and were associated with patients' prognosis in the human breast cancer datasets.

**Conclusion:**

The signature-based cell cycle analysis method presented in this report, would potentially be of value for cancer characterization and diagnostics.

## Background

A fundamental characteristic of all cancers is cell cycle deregulation [1]. Although diverse factors such as point mutation, gene amplification, activation of oncogenes, inactivation of tumor suppressors, and hypermethylation are involved in cancer development, their influence ultimately is on the cell cycle machinery. Therefore, various methods of cell cycle phase estimation have been developed. The M phase indicator mitotic index, the number of mitotic bodies in a microscopic field, and the S-phase fraction, a DNA flow cytometry determination, are used to measure the tumor proliferation rate and are predictive for breast cancer prognosis [2-4]. Immunohistochemistry (IHC) against cell cycle markers is another tool. For example, the expression of G1-S transition marker *cyclin E*, S-G2 marker *cyclin A*, or S-G2-M marker *geminin *are predictive of poor prognosis of breast cancers [2-5]. However, these methods rely on one or few measurements and consequently provide a limited scope of information. There is a need for more systematic methods of cell cycle phase analysis, such as microarray-based techniques [3,4].

Gene expression signatures, which are capable of predicting the state of a sample from a given microarray dataset, are the emerging technology for developing cancer therapeutics. The "70-gene signature" from a breast cancer dataset has shown predictive power for the risk of recurrence [6]. The "pathway deregulation signature" has shown the ability to predict pathway status and to characterize breast, lung and ovarian cancers [7]. The "chemotherapy response signature" has accurately predicted clinical response to cytotoxic drugs for breast and ovarian cancers [8]. Here, we report the development of the "cell cycle signature (CCS)" which indexes the cell cycle phase distribution from microarray profiles considering both cycling and non-cycling cells. The CCS method depicted "buried" cell cycle phase distributions that were oncogenic-event specific in a mouse tumor model dataset and were associated with patients' prognosis in human breast cancer datasets. The method has a potential to be of value in the characterization and diagnosis of cancers.

## Results

### Algorithm

**Figure 1:**
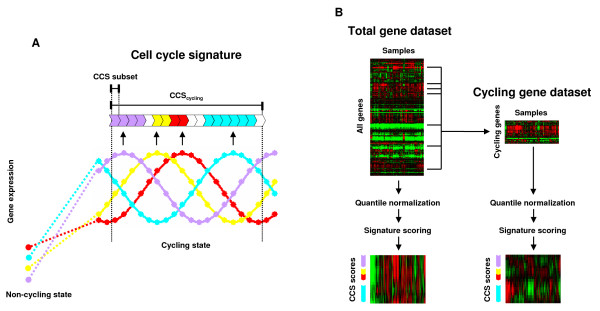
**Flow diagram of the cell cycle signature (CCS) method**. (A) CCS_cycling _consists of genes which preferentially express in cycling cells and in a cell cycle-regulated manner, representing the entire cell cycle. Each CCS subset consists of genes whose expressions peak at specific stages of the cell cycle, representing the corresponding stages. (B) From the given total gene dataset, the cycling gene dataset is created by extracting the expression values of CCS_cycling _genes. Both datasets are independently quantile normalized and the CCS scores are calculated for each.

To analyze cell cycle phase distribution, a series of CCSs were created as described in Methods (Fig. 1A, Additional file 1). The CCS masterset, 252 genes that express preferentially in cycling cells and in a cell cycle-regulated manner, represents the entire cell cycle and is henceforth denoted as CCS_cycling_. Eighteen CCS subsets, each composed of genes whose expressions peak at a specific stage of the cell cycle, represent the phases of the cell cycle and are denoted using the subscript naming convention of CCS_phase_. For example, the CCS subsets for the G1 phase are expressed as CCS_G1_, for the G2-M phase as CCS_G2-M_, and so on.

Solid tumors are composed of various proportions of cycling and non-cycling cells [9], and cell cycle phase distributions can be assessed as per total cells or as per cycling cells. Since microarray measurements are the net expression of all cells in the sample, the data is generally per total cells. To obtain data per cycling cells from a given microarray dataset (Fig. 1B, total gene dataset), a subdataset is created by extracting the expression values of CCS_cycling _genes (Fig. 1B, cycling gene dataset). Then, both the total and the cycling gene datasets undergo quantile normalization which gives the same expression value distribution for each sample [10]. In the total gene dataset, normalization is done on all genes. On the other hand, in the cycling gene dataset, normalization is done only on the cycling genes. Because genes in the CCS_cycling _preferentially express in cycling cells, the influence of non-cycling cells would be limited for the cycling gene dataset. Scores for each CCS are calculated for both datasets. CCS_cycling _and CCS_phase _scores for the total gene dataset could index the proportion of cycling cells and of cells at the designated cell cycle phase per total cells, respectively. Similarly, CCS_phase _scores for the cycling gene dataset could index the proportion of cells at the cell cycle phase per cycling cells. CCS_cycling _scores for the cycling gene dataset could index the proportion of cycling cells per cycling cells and thus would show constant values.

### Validation

**Figure 2:**
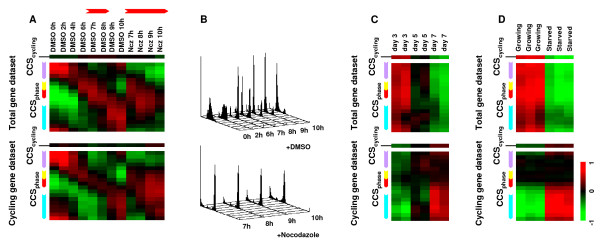
**Validation of the CCS method using datasets of the HCT116 cell cycle and quiescence-induced cells**. (A) CCS score heat maps for the HCT116 cell cycle dataset. Synchronized HCT116 cells were profiled at 0, 2, 4, 6, 7, 8, 9 and 10 h after release (DMSO, 0–10 h). Nocodazole-treated cells were profiled in parallel (Ncz, 7–10 h). CCS scores were calculated for both the total (upper panel) and the cycling (lower panel) gene dataset. Each column represents an experimental sample and each row a CCS subset. Cell cycle phases for CCS are indicated by the colored bars on the left of each map (G1; cyan, S; purple, G2; yellow, and M; red). Red bars above the columns indicate estimated M phase. (B) Flow cytometric analysis of HCT116 cells. Synchronized HCT116 cells were monitored by DNA flow cytometry after release with DMSO (upper panel) or nocodazole (lower panel). (C) CCS score heat maps for the Fournier *et al*. dataset of HMECs grown in 3D culture. In this system, rapidly growing HMECs (day 3) enter the quiescent state over several days (day 7). (D) CCS score heat maps for the Cam *et al*. dataset of T98 breast cancer cells. The profiles of growing and serum-starved cells for 3 days were analyzed.

In the preliminary analysis of the Whitfiled *et al*. cell cycle dataset [11], CCS indexed cell cycle phase distribution as expected (Additional file 2). To confirm that the CCS method is valid for independent datasets, a cell cycle dataset of synchronized HCT116 cells was prepared and analyzed. As shown in Fig. 2A, similar heat map patterns were observed for the total and the cycling gene datasets. Differences in the CCS_cycling _scores for both the total and the cycling gene datasets were slight in the situation where most cells were expected to be in the cell cycle. Peaks in the CCS_phase _scores shifted according to cell cycle progression (Fig. 2A, DMSO 0–10 h), and peaks ceased around the M phase in cells treated with the mitosis inhibitor nocodazole (Fig. 2A, Ncz 7–10 h), consistent with DNA flow cytometry measurements (Fig. 2B). The CCS method was able to index cell cycle phase distribution even for an independent cell cycle dataset derived from a different cell line and a different platform.

Solid tumors are not solely composed of cycling cells but contain various numbers of non-cycling cells [9]. Theoretically, changes in the proportion of cycling cells in the sample are expected to evenly change the proportion of cells in all cell cycle phases. To examine the influence of changes in the proportion of cycling cells on CCS scores, analysis was conducted on the Fournier *et al*. dataset [12] of profiles of human mammary epithelial cells (HMECs) cultured in leucine-rich extra cellular matrix. In this system, HMECs grow exponentially and then enter a quiescent state [12,13]. As shown in Fig. 2C, CCS_cycling _and CCS_phase _scores for the total gene dataset uniformly decreased as the HMECs transitioned from cycling (day 3) to non-cycling state (day 7) (Fig. 2C, upper panel). According to the DNA flow cytometry estimation in the original report, the S phase and G2+M phase fraction size decreased from 15% ± 5.1 (day 5) to 5.5% ± 0.5 (day 7), and from 12% ± 1.1 (day 5) to 7% ± 2.5 (day 7), respectively (day 3 data was not available) [12]. On the other hand, the G0+G1 phase fraction size increased from 73% ± 6.3 (day 5) to 86% ± 4.6 (day 7). Due to the inability of DNA flow cytometry to distinguish cells in G0 from cells in G1, decisive conclusions cannot be made. However, from two situations in which 1) 3D cultured HMECs gradually underwent growth arrest and 2) CCS_G1 _scores decreased at day 7, this increase can be regarded as an increase in the number of cells at the G0 phase as well as a decrease in the number of cells at the G1 phase. To our surprise, the heat map for the cycling gene dataset showed increasing CCS_G1 _scores towards day 7 (Fig. 2C, lower panel). This increase in CCS_G1 _scores could be due to the G1 phase prolongation which is known to occur under G0-inducing conditions, such as serum starvation and development [14,15]. For further confirmation, we analyzed the Cam *et al*. dataset [16] of profiles of growing and serum starved T98 breast cancer cells. Similar to the results for HMECs, a uniform decrease in CCS_cycling _and CCS_phase _scores for the total gene dataset was observed in serum-starved cells (Fig. 2D, upper panel). In addition, an increase in CCS_G1 _scores for the cycling gene dataset was observed (Fig. 2D, lower panel), indicating prolongation of the G1 phase. Taken together, these results suggested that changes in the proportion of cycling cells in the sample can be presented as uniform changes in CCS_cycling _and CCS_phase _scores for the total gene dataset.

The mammalian cell cycle is a highly regulated and conserved process [17]. To investigate whether CCS derived from human datasets can be used to closely related species, the Yamamoto *et al*. dataset [18], cell cycle profiles (G0 to S) of NIH3T3 mouse fibroblasts, was analyzed. The heat map showed changes in the proportion of cycling cells (Additional file 3: upper panel) as well as cell cycle progression from G1 to S phase (Additional file 3: lower panel), as quiescent cells (FGF 0 h) re-enter the cell cycle, progress through G1 phase and enter S phase (FGF 12 h). These results showed that the human CCS created in this study can be applied for the analysis of mouse datasets.

### Analysis on mouse tumor model dataset

**Figure 3:**
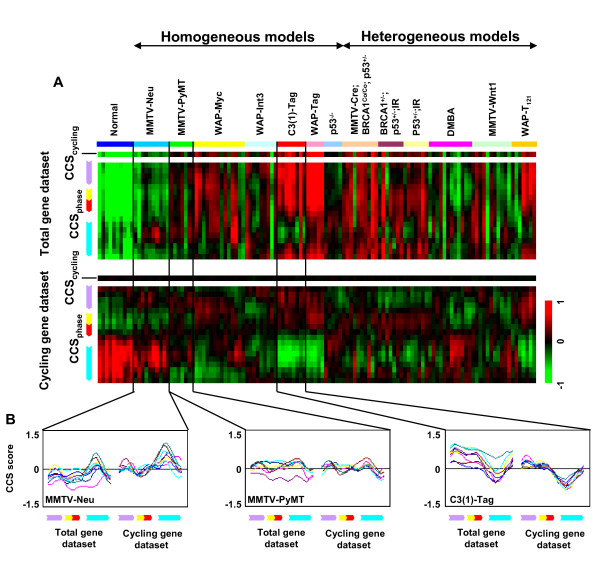
**Analysis of the Herschkowitz *et al*. mouse tumor model dataset**. (A) CCS score heat maps for the Herschkowitz *et al*. dataset. 122 profiles from 13 mouse tumor models and normal samples were analyzed. Tumors are aligned according to the homogeneous-heterogeneous classification of Herschkowitz *et al*. (B) CCS score plots for selected homogeneous models. CCS_phase _scores of the MMTV-Neu, MMTV-PyMT and C3(1)-Tag models were plotted. X axis represents cell cycle phases and Y axis represents magnitude of CCS score.

The CCS method was applied to the Herschkowitz *et al*. dataset [19] which contains 122 profiles of 13 different mouse mammary carcinoma models and normal samples. The authors reported that some models developed similar tumors (homogeneous models) of gene expression and histological phenotype while other models showed heterogeneity (heterogeneous models) and gave "randomness of the molecular basis of tumor initiation" as the reason for the heterogeneity. As shown in Fig. 3A, CCS_cycling _and CCS_phase _scores for the total gene dataset for the normal samples were consistently very low, while scores for tumors were varying degrees higher, indicating variation in the proportion of cycling cells. It is reasonable that heterogeneous models show variation in CCS_cycling _and CCS_phase _scores. However, variation was also seen in each homogeneous model, although Tag models had a tendency towards higher scores and the Neu model had a tendency towards lower scores. In contrast, CCS_phase _scores for the cycling gene dataset were similar within the same homogeneous models, except in the Myc model (Fig. 3A, lower panel). To illustrate this in detail, CCS_phase _scores of several models for both datasets were plotted as shown in Fig. 3B. It can be seen that each model has a specific cell cycle phase distribution. High CCS_G1 _and low CCS_S-G2-M _scores were seen in the Neu model. The opposite pattern was seen in one of the Tag models. The Myc model showed two different cell cycle phase distributions (Additional file 4) and the reason is not clear. However, because Myc has been reported to induce genomic instability and to contribute to tumorigenesis through a dominant mutator effect [20], additional oncogenic events may have been induced. In all cases, plots for the total gene dataset were vertically shifted in varying degrees which would be due to the influence of non-cycling cells, as presented in HMECs and T98 cells. On the other hand, plots for the cycling gene dataset showed minimal variation in alignment. These results indicated two findings: (*i*) the cell cycle phase distribution reflects the oncogenic events in tumors, and (*ii*) the cell cycle phase distribution can be better indexed when the influence of non-cycling cells is taken into account. The advantage of the CCS method can be underscored considering that the current cell cycle phase estimation methods relying on one or few measurements are not sufficient to depict cell cycle phase distribution or to distinguish non-cycling cells.

### Analysis on human breast cancer datasets

**Figure 4:**
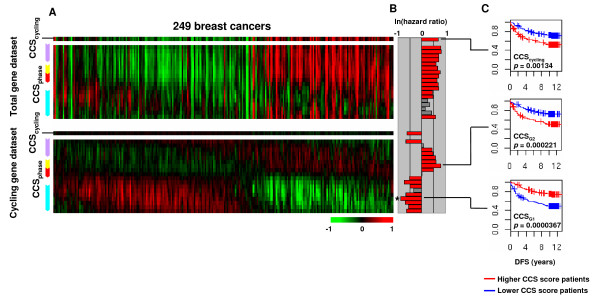
**Analysis of the Ivshina *et al*. human breast cancer dataset**. (A) CCS score heat maps for the Ivshina *et al*. dataset. Patients were aligned by the peak in CCS_phase _scores for the cycling gene dataset. (B) Prognostic values of each CCS for disease free survival (DFS). Patients were dichotomized by the median of each CCS score and risk differences of two groups for DFS were assessed by log-lank test and Cox univariate analysis. Log scale hazard ratios are indicated by the colored bars: log-rank *p *< 0.05 (red), and *p *≥ 0.05 (gray). The highest prognostic value is indicated by (*). (C) Survival curves for selected signatures. Higher CCS score patients (blue); lower CCS score patients (red).

The CCS method was applied to the Ivshina *et al*. dataset [21] from a panel of 249 human breast cancers. The heat map for the total gene dataset showed various CCS_cycling _scores, indicative of variations in the proportion of cycling cells in the sample (Fig. 4A, upper panel). The CCS_phase _scores were not uniformly changed in some patients, suggesting that cell cycle phase distributions were also altered. The heat map for the cycling gene dataset displayed a rolling wave pattern (Fig. 4A, lower panel). Patients with high CCS_cycling _scores for the total gene dataset had high CCS_S-G2-M _and low CCS_G1 _scores for the cycling gene dataset, but several exceptions existed (Fig. 4A), reminding the influence of non-cycling cells found in the analysis of mouse tumor models. Clinical annotations were available for this dataset and so the relevance between CCS scores and patient prognosis were tested. Patients were dichotomized by the median of each CCS score and then the risk differences between the two groups for disease free survival (DFS) were assessed using log-rank test and Cox univariate analysis (Fig. 4B). The CCS_cycling _score for the total gene dataset was significantly predictive of poor prognosis (Hazard ratio [HR] = 1.98, *p *= 0.00134) (Fig. 4B and Fig. 4C, CCS_cycling_), consistent with the common view that a larger number of cycling cells correlates with worse clinical outcome. The CCS_S-G2-M _and several CCS_G1 _scores for the total gene dataset were also predictive of poor prognosis. On the other hand, CCS_G1 _scores for the cycling gene dataset had an adverse prognostic power and gave the highest prognostic value among the tests (HR = 0.41, *p *= 0.0000367) (Fig. 4B and Fig. 4C, CCS_G1_).

To exclude the possibility of dataset specificity, the CCS method was also applied to the Langerød *et al*. dataset [22] from a panel of 80 breast cancers. Similar results were obtained (Additional file 5). For the total gene dataset, variations in CCS_cycling _scores and non-uniform changes in CCS_phase _scores in some patients were observed. Patients with high CCS_cycling _scores for the total gene dataset had high CCS_S-G2-M _and low CCS_G1 _scores for the cycling gene dataset with some exceptions. CCS_G1 _scores for the cycling gene dataset were predictive for DFS as with the Ivshina *et al*. dataset and gave the highest prognostic value (HR = 0.41, *p *= 0.00553) (Additional file 5). Taken together, these results indicated that: (*i*) variations in the proportion of cycling cells exist among tumors, (*ii*) the proportion of cycling cells correlated to the cell cycle phase distribution per cycling cells with several exceptions, and (*iii*) the cell cycle phase distribution per cycling cells better associated with patients' prognosis.

## Discussion and conclusion

In this study, we developed a signature-based method to index cell cycle phase distribution from microarray profiles under consideration of cycling and non-cycling cells, providing two sources of valuable information on cancers.

One source of information is the proportion of cycling cells in the sample. The rationale of most current cell cycle phase estimation methods, including mitotic index, S phase fraction and IHC against cell cycle markers, is that the high proliferative tumors leading to poor prognosis contain more cycling cells. In the analysis of the human breast cancer datasets, higher CCS_cycling _scores for the total gene dataset, indicative of a larger number of cycling cells in the sample, did associate with poor prognosis. Naturally, it can be thought that an increase in the number of cycling cells leads to a uniform increase in the number of cells at all cell cycle phases. However, some patients showed non-uniform changes in CCS_phase _scores for the total gene dataset (Fig. 4A, upper panel), suggesting that each cell cycle phase was not evenly changed. Similarly, Whitfield *et al*. observed that some cell cycle-regulated genes did not express in correlation with proliferation status in some breast cancers [11]. Furthermore, although the G1 phase is a part of the cell cycle, G1 phase marker *cyclin D1 *often negatively correlates with poor prognosis of breast cancers [2-4,23]. Therefore, considering only the proportion of cycling cells seems insufficient.

The other source of information is cell cycle phase distribution. A number of oncogenic events are known to perturb the duration of cell cycle phases. For example, activation of oncogenes such as *v-H-ras*, *v-Src*, *v-Raf*, *cyclin D1*, *cyclin E*, and *c-myc *shortens the G1 phase [24-26]. Loss of tumor suppressor *Pten *shortens the G1 phase [27] and loss of *Lzts1 *and *Lats2 *shortens the M phase [28,29]. Viral infections such as SV40-Tag and HTLV-1 Tax also shorten the G1 phase [30,31]. Such perturbations in the cell cycle phase duration subsequently alter the cell cycle phase distribution. Thus, the cell cycle phase distribution per cycling cells would reflect the biology of cancers. Actually, in the analysis of mouse tumor models, oncogenic-event specific cell cycle phase distributions were observed. This suggests that the cell cycle phase distribution under consideration of both cycling and non-cycling cells has a potential for cancer characterization.

**Figure 5:**
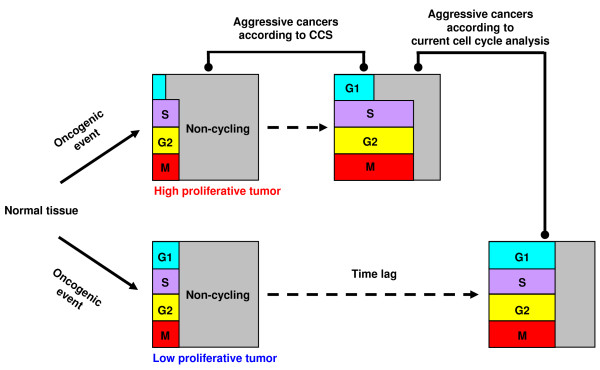
**A model of tumors with different cell cycle phase distributions**. Oncogenic events perturb the cell cycle each in their unique way, which alters cell cycle phase distribution as well as proliferation rate. High proliferative tumors grow rapidly and produce large number of cycling cells, but exceptions exist at a low probability. CCS can characterize them under consideration of cycling and non-cycling cells.

A model of tumors with different cell cycle phase distributions is proposed in Fig. 5. Oncogenic events perturb the cell cycle each in a unique way which in turn alters the cell cycle phase distribution as well as the proliferation rate. High proliferative tumors grow rapidly and thereby produce a large number of cycling cells. The opposite is the true for low proliferative tumors. However, high proliferative tumors with a small number of cycling cells or low proliferative tumors with a large number of cycling cells would exist at a low probability. This model would account for non-uniform changes in CCS_phase _scores for the total gene dataset found in some breast cancer patients, the Whitfield *et al*.'s observation, and the adverse prognostic value of *cyclin D1*. Current cell cycle phase estimation methods are insufficient for detecting such cancers. Mitotic index and S-phase fraction do not recognize non-cycling cells. Combinatorial IHC [32] still needs improvement and validation. Shetty *et al*. reported a relationship between breast cancer grade and G1 phase length estimated from the ratio of *geminin *and *Ki67 *IHC measurements; however, it was not significant [33]. The CCS method, on the other hand, indexed the cell cycle phase distribution under consideration of cycling and non-cycling cells, and showed a potential for characterizing cancers.

Previously, as an alternative microarray-based cell cycle analysis technique, Lu *et al*. introduced the "expression deconvolution" method [34]. To predict the cell cycle phase distribution of yeast, they prepared about 700 equations with 5 variables representing 5 cell cycle phases and searched for the optimal solution. The method has comparable or even better potential to improve cancer characterization than the CCS method. However, it requires a tremendous amount of computational resources to find the optimal solution and avoid the local minimum, especially as the number of variables increases (18 + 1 phases were analyzed in our study). There are some hurdles that need to be overcome before high resolution cell cycle phase analysis is practical and we are currently tackling some of them.

## Methods

### Cell Culture and Synchronization

The HCT116 colorectal cancer cell line (ATCC) was grown in McCoy's 5A medium modified (Sigma-Aldrich) with 10% FBS (JBS) and maintained at 37°C and 5% CO_2_. Synchronous culture was obtained by incubating cells for 19 h in 2 mM of thymidine, followed by a 9-h incubation in normal medium and a second 16-h incubation in thymidine (2 mM). Cells were washed with normal medium followed by treatment with DMSO for 0, 2, 4, 6, 7, 8, 9, and 10 h as a control or 0.1 mg/ml nocodazole (Sigma-Aldrich) for 7, 8, 9, and 10 h. Cells were stained with propidium iodide and analyzed with DNA flow cytometry.

### Microarray

Total RNA was reverse transcribed, labeled, and hybridized to Human Genome U133 Plus 2.0 arrays (Affymetrix) according to the manufacturer's instructions. The expression value for each probe was calculated using the GC-RMA algorithm. The microarray data were deposited in the GEO database (GEO number: GSE14103).

### Signature development

Two datasets were used to create the CCS. First, the Whitfield *et al*. dataset [11] of 47 profiles of synchronized Hela S3 cells for 0–46 h time points (1-h intervals) after release of double thymidine block was analyzed to identify genes which express in a cell cycle-regulated manner. Raw signal intensities from the Cy5 and Cy3 channels were quantile normalized for each sample. Cy5/Cy3 ratios were log-transformed and quantile normalized across the arrays. Resulting values were smoothened using a moving average with a window size of 3 and were standardized by Z-transformation. Then, Fourier transformations were applied to each probe for 1-40-h periods in 15-min increments to identify periodicity and phase offset. Fourier transformation magnitudes for the known 51 cell cycle-regulated genes (listed in Whitfield *et al*. [11]) demonstrated a peak at the 14.75-h periodicity (Additional file 6). Thus, probes were selected using the criterion of

Z-score(*P*_*i*_) > 1.96

where *P*_*i *_is the Fourier transformation magnitude of the 14.75-h periodicity for probe *i*, *i *= 1,..., 44,160. The analysis yielded a list of 1,633 periodically expressed probes representing 976 genes. Second, the Bar-Joseph *et al*. dataset [35] of 17 profiles of synchronized primary human foreskin fibroblasts (FFs) for 0–32 h time points (2-h intervals) after release of double thymidine block and 2 profiles of serum starved FFs was investigated to identify genes which preferentially express in cycling cells. Serum starved cells are known to exit the cell cycle phase and to enter the non-cycling G0 phase [14], thus probes, whose expression is constantly higher throughout the cell cycle compared with non-cycling cells, were selected by the criterion

max(*e*_*ij*_) < min(*e*_*ik*_)

where *e*_*ij *_is the expression value for probe *i *of serum-starved FFs sample *j*, *j *= 1, 2, and *e*_*ik *_is the expression value for probe *i *of the synchronized FFs sample *k*, *k *= 1,..., 17. This yielded 2,304 out of 22,277 probes representing 1,779 genes. Then, from the intersection, a list of 335 probes representing 252 genes was obtained. These genes which preferentially express in cycling cells and in a cell cycle-regulated manner compose the CCS masterset (CCS_cycling_). A number of well-known proliferation markers such as *Ki67*, *geminin*, *TOP2A*, *aurora A*, and *PCNA *[1-5,32] were included in this signature, while some cell cycle-regulated genes such as *p21 *and *cyclin G1 *whose expression can be up-regulated in non-cycling cells [36,37] were not. Lastly, according to their phase offsets, probes for CCS_cycling _were assigned to 18 CCS subsets (CCS_phase_) which correspond to a 360° cell cycle evenly divided into 20° increments, so that each CCS subset contains at least 3 genes. Because some genes were represented by multiple probes, the same genes may appear in different CCS subsets. The CCS gene list is shown in Additional file 1.

### Signature scoring and data visualization

The given microarray dataset was used as the total gene dataset. The cycling gene dataset was created by extracting the expression values for CCS_cycling _constituents from the total gene dataset. Both total and cycling gene datasets then underwent the following steps independently to give CCS scores. Expression values were log-transformed, quantile normalized to achieve the same expression value distribution for each sample, and standardized with Z-transformation across the samples. The Z-scores of the probes for each CCS genes were averaged for each sample and used as the CCS scores. To obtain robust scores, each CCS_phase _score was adjusted by averaging with the neighboring CCS scores twice for a total of two cell cycle rounds. Heat maps were created by "Java Treeview" [38]. In the analysis of the mouse tumor model dataset, gene ID mapping was done using human-mouse orthology information from HomoloGene [39]. In the analysis of human breast cancer datasets, patients were ordered by peak in CCS_phase _scores for the cycling gene dataset.

### Survival analysis

Patients were dichotomized by the median of each CCS score. To assess the risk difference between two groups for DFS, Kaplan-Meier survival analysis, log-rank test and Cox univariate analysis were conducted using R "survival" package.

## Authors' contributions

HM and KK designed the research. HM and YN performed the research. HM, NI, AS and KK participated in writing the manuscript. All authors read and approved the final manuscript.

## Supplementary Material

Additional file 1 **The gene list for cell cycle signatures. **The CCS genes and assigned CCS subset IDs are listed. Click here for file

Additional file 2 **Validation of CCS method in the Whitfiled *et al*. cell cycle dataset. **CCS scores were calculated for the total (upper panel) and the cycling (lower panel) gene dataset. The purple bars above the columns indicate Whitfield *et al*.'s estimations of the S phase. Click here for file

Additional file 3 **Analysis of the Yamamoto *et al*. dataset. **Serum starved NIH3T3 cells were stimulated with FGF to re-enter the cell cycle. Profiles of unstimulated cells (FGF 0 h) and FGF-stimulated cells (FGF 3–12 h) were analyzed. Click here for file

Additional file 4 **CCS score plots for the WAP-Myc model. **Same as for Fig. 3B. Click here for file

Additional file 5 **Analysis of the Langerød *et al*. breast cancer dataset. **(A), (B) and (C) are the same as in Fig. 4. Click here for file

Additional file 6 **Power spectrum of the 51 cell cycle-regulated genes. **The Hela S3 cell cycle dataset was processed as described in Methods. Fourier transformation magnitudes for the known 51 cell cycle-regulated genes for each periodicity were averaged and plotted. Click here for file
