# Supplementary figures and images for: A signature-based method for indexing cell cycle phase distribution from microarray profiles
Source: BMC Genomics. 2009 Mar 30;10:137. doi: 10.1186/1471-2164-10-137 (PMC2676301; doi:10.1186/1471-2164-10-137)

## Slide 1
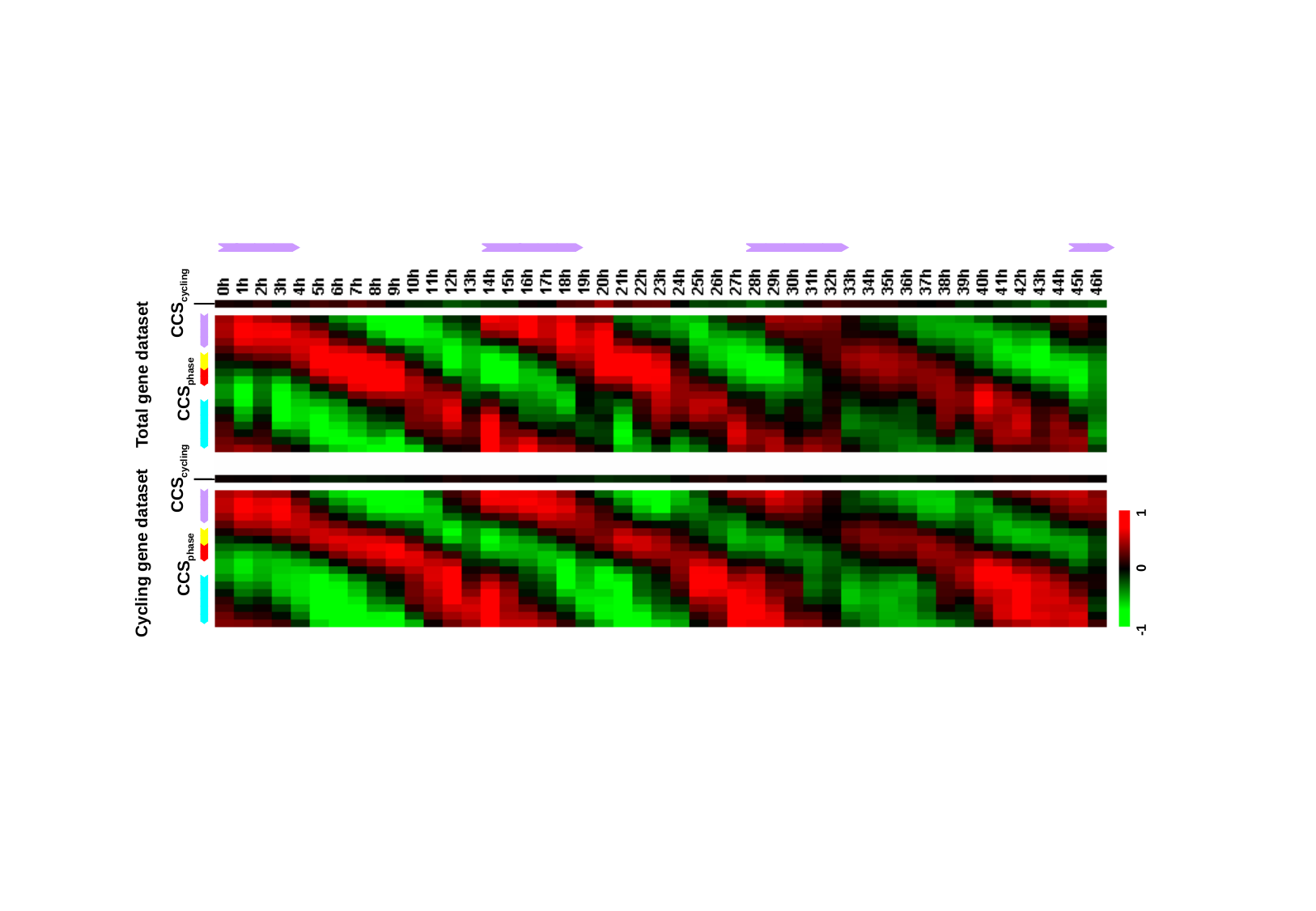

CCScycling
CCSphase
Total gene dataset
CCScycling
CCSphase
Cycling gene dataset
1
0
-1

Supplement: Additional file 2 — Validation of CCS method in the Whitfiled et al. cell cycle dataset. CCS scores were calculated for the total (upper panel) and the cycling (lower panel) gene dataset. The purple bars above the columns indicate Whitfield et al.'s estimations of the S phase. [file 1471-2164-10-137-S2.ppt]

## Slide 1
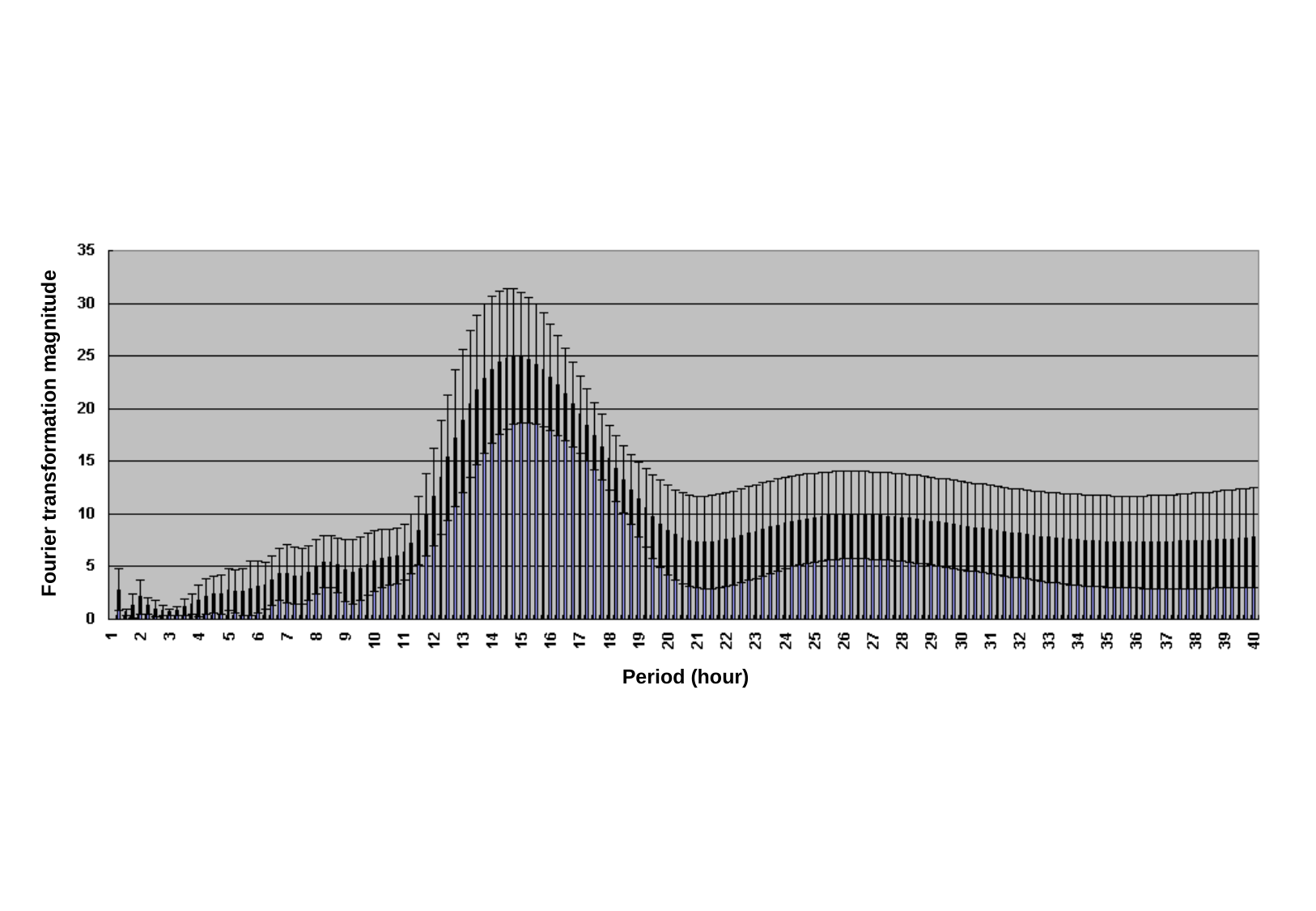

Fourier transformation magnitude
Period (hour)

Supplement: Additional file 6 — Power spectrum of the 51 cell cycle-regulated genes. The Hela S3 cell cycle dataset was processed as described in Methods. Fourier transformation magnitudes for the known 51 cell cycle-regulated genes for each periodicity were averaged and plotted. [file 1471-2164-10-137-S6.ppt]
