# Supplementary material for: A signature-based method for indexing cell cycle phase distribution from microarray profiles
Source: BMC Genomics. 2009 Mar 30;10:137. doi: 10.1186/1471-2164-10-137 (PMC2676301; doi:10.1186/1471-2164-10-137)
Supplement: Additional file 3 — Analysis of the Yamamoto et al. dataset. Serum starved NIH3T3 cells were stimulated with FGF to re-enter the cell cycle. Profiles of unstimulated cells (FGF 0 h) and FGF-stimulated cells (FGF 3–12 h) were analyzed. [file 1471-2164-10-137-S3.ppt]

## Slide 1
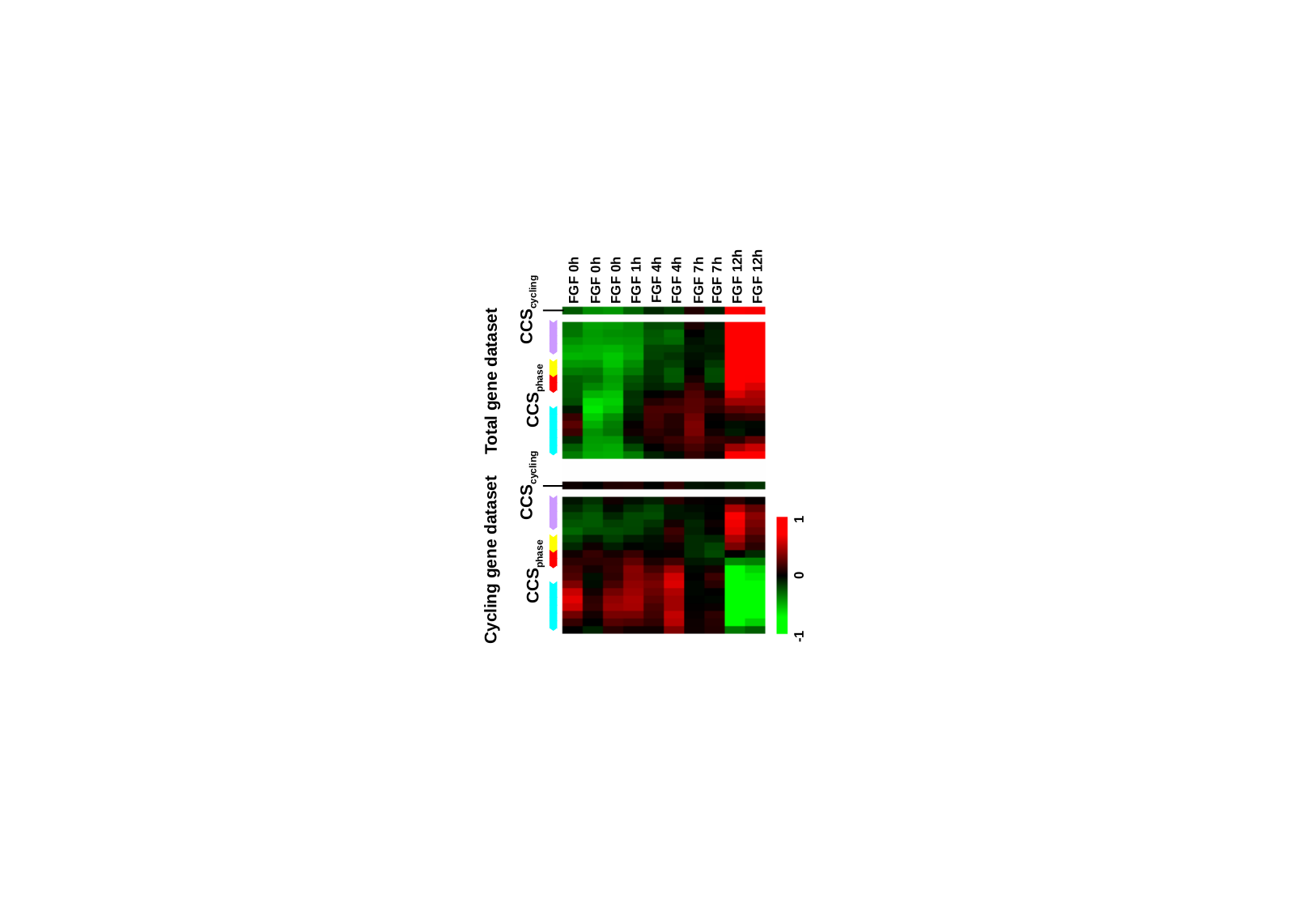

FGF 4h
CCScycling
CCSphase
Total gene dataset
CCScycling
CCSphase
Cycling gene dataset
FGF 0h
FGF 0h
FGF 0h
FGF 1h
FGF 4h
FGF 7h
FGF 7h
FGF 12h
FGF 12h
1
0
-1
