# Supplementary material for: A signature-based method for indexing cell cycle phase distribution from microarray profiles
Source: BMC Genomics. 2009 Mar 30;10:137. doi: 10.1186/1471-2164-10-137 (PMC2676301; doi:10.1186/1471-2164-10-137)
Supplement: Additional file 4 — CCS score plots for the WAP-Myc model. Same as for Fig. 3B. [file 1471-2164-10-137-S4.ppt]

## Slide 1
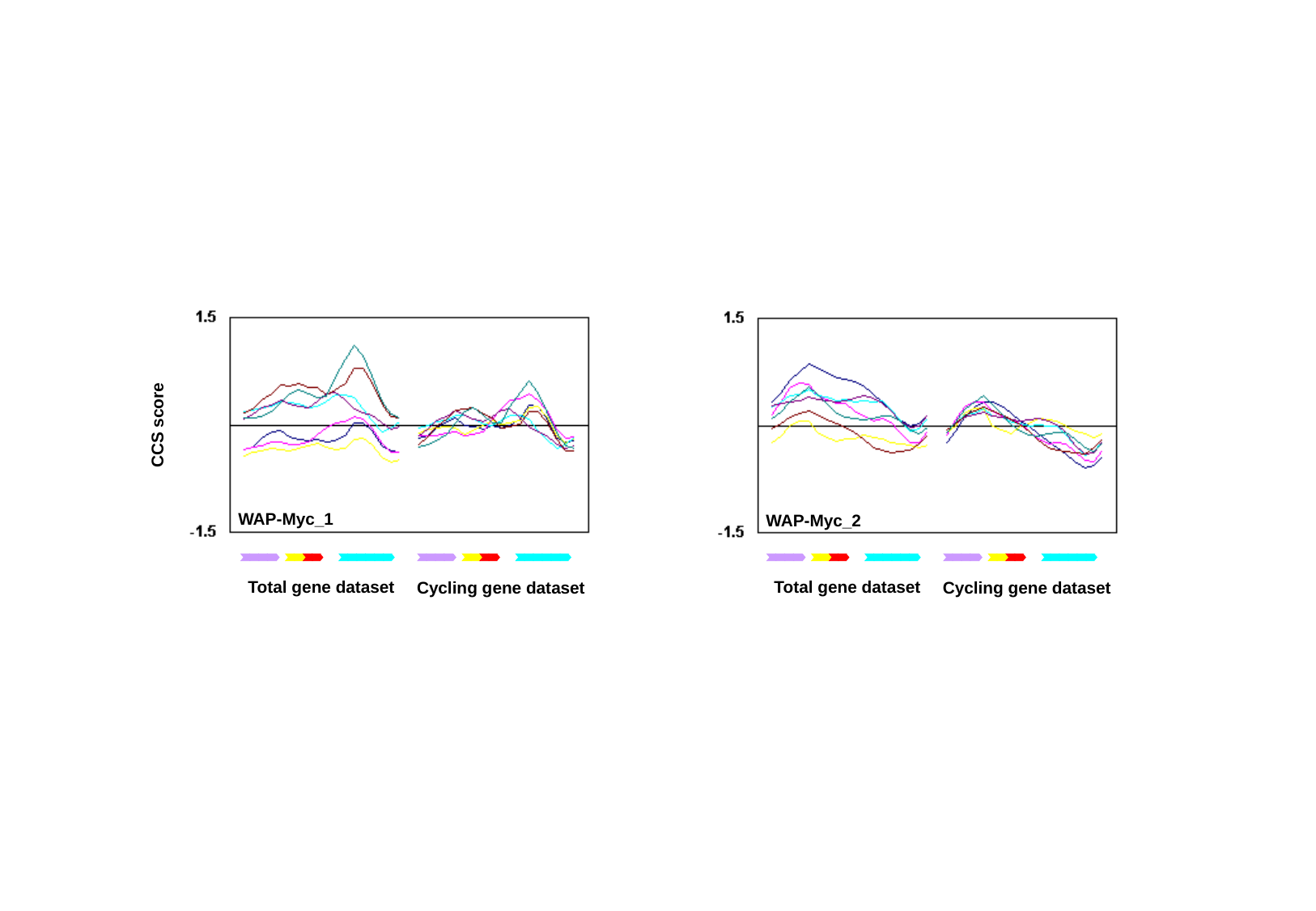

CCS score
WAP-Myc_1
WAP-Myc_2
Total gene dataset
 Cycling gene dataset
Total gene dataset
 Cycling gene dataset
