# Supplementary material for: A signature-based method for indexing cell cycle phase distribution from microarray profiles
Source: BMC Genomics. 2009 Mar 30;10:137. doi: 10.1186/1471-2164-10-137 (PMC2676301; doi:10.1186/1471-2164-10-137)
Supplement: Additional file 5 — Analysis of the Langerød et al. breast cancer dataset. (A), (B) and (C) are the same as in Fig. 4. [file 1471-2164-10-137-S5.ppt]

## Slide 1
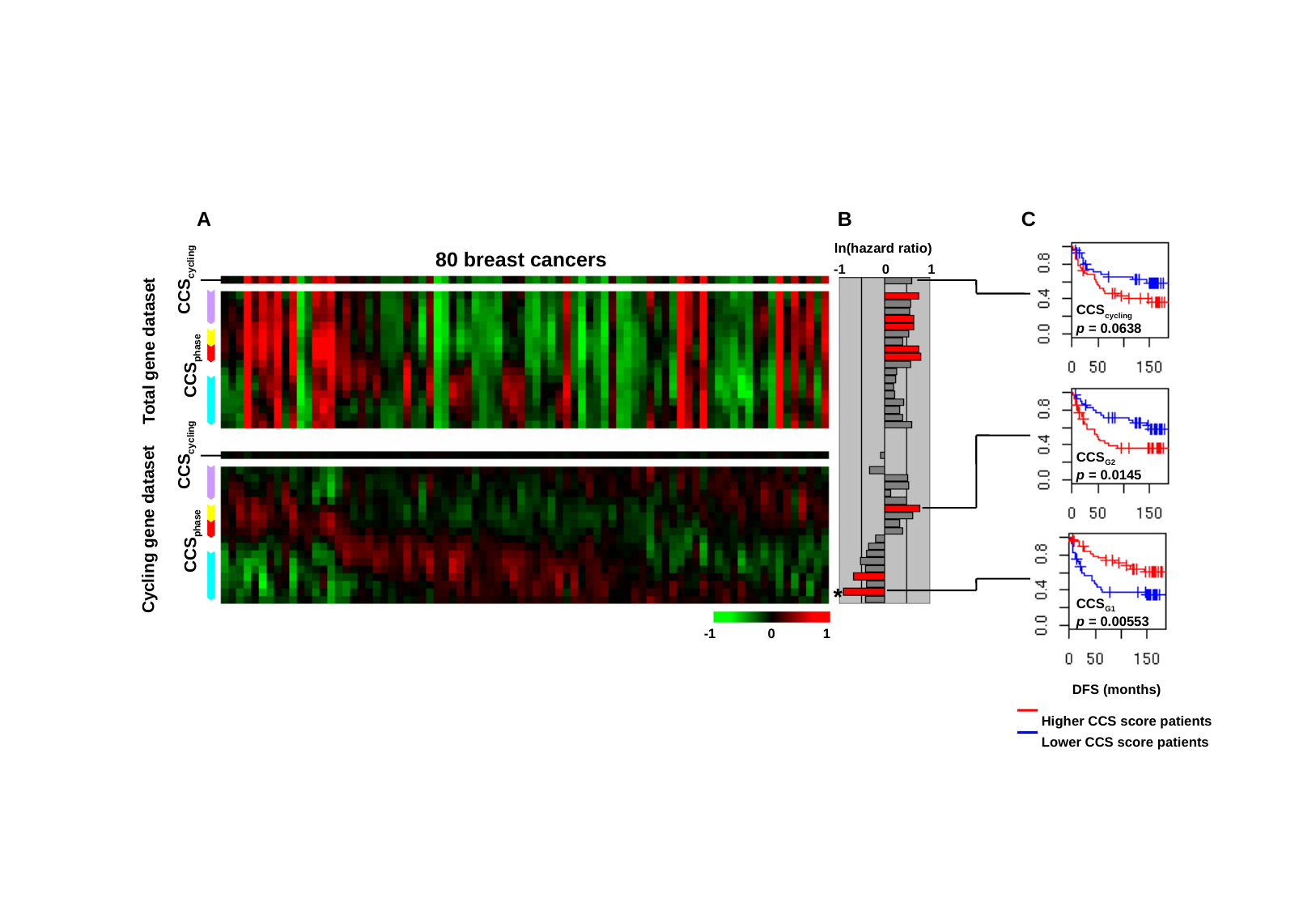

A
B
C
CCScycling
CCSphase
Total gene dataset
CCScycling
CCSphase
Cycling gene dataset
ln(hazard ratio)
80 breast cancers
-1
0
1
CCScycling
p = 0.0638
CCSG2
p = 0.0145
*
*
CCSG1
p = 0.00553
-1
0
1
DFS (months)
Higher CCS score patients
Lower CCS score patients
